# A New Criterion for Shear Wave Elastometric Assessment Using Modulus of Stiffness Difference between Object and Environment

**DOI:** 10.17691/stm2022.14.5.01

**Published:** 2022-09-29

**Authors:** I.Yu. Demin, P.I. Rykhtik, А.E. Spivak, D.V. Safonov

**Affiliations:** Associate Professor, Department of Acoustics, Faculty of Radiophysics; National Research Lobachevsky State University of Nizhni Novgorod, 23 Prospekt Gagarina, Nizhny Novgorod, 603950, Russia;; Head of the Department of Radiation Diagnostics; Volga District Medical Center of the Federal Medical and Biological Agency of Russia, 2 Nizhne-Volzhskaya naberezhnaya, Nizhny Novgorod, 603001, Russia;; PhD Student, Faculty of Radiophysics; National Research Lobachevsky State University of Nizhni Novgorod, 23 Prospekt Gagarina, Nizhny Novgorod, 603950, Russia;; Professor, Head of the Department of Radiation Diagnostics, Faculty of Advanced Medical Training; Privolzhsky Research Medical University, 10/1 Minin and Pozharsky Square, Nizhny Novgorod, 603005, Russia

**Keywords:** shear wave elastography, ultrasound elastometry, elastographic phantom

## Abstract

Ultrasound shear wave elastography is a modern method that is based on measuring the shear wave velocity making it possible to determine stiffness of soft biological tissues at an arbitrary point (point elastography) or to construct a two-dimensional color image with a subsequent point measurement of stiffness (two-dimensional elastography) and therefore to compare the stiffness of an object with a medium or objects with each other.

**The aim of the study** is to develop a new criterion for the comparative assessment of objects with different stiffness during shear wave elastometry: modulus of stiffness difference between object and environment.

**Materials and Methods:**

Using the original technology of building two-dimensional color elastogram, point and two-dimensional shear wave elastography were performed using linear sensors on commercial ultrasound scanners: Aixplorer (SuperSonic Imagine, France), Acuson S2000 (Siemens, Germany), and Verasonics acoustic system (Verasonics Inc., USA) with an open architecture to determine the stiffness values of focal inclusions and compare them with each other with the help of a new comparative elastomeric assessment criterion: modulus of stiffness difference between object and environment. First, the accuracy of the scanners under test was compared on a calibrated Elasticity QA Phantom, model 049 (Computerized Imaging Reference Systems Company, USA) with a known stiffness of various inclusions and thereafter on an uncalibrated BP1901 phantom (Blue Phantom, USA) with unknown stiffness of inclusions. The obtained values were compared to determine the influence of subjective factors on the measurement results.

**Results:**

To assess the stiffness of the foci and compare the values with each other taking into account the rigidity of the environment, it is proposed to use a new criterion for the comparative assessment — the modulus of stiffness difference between focus and environment, which quantitatively characterizes the difference between these values. According to this criterion, all three ultrasound scanners have been established to show high and comparable accuracy in determining the stiffness of inclusions within the homogeneous medium in the experiments on phantoms. Two-dimensional shear wave elastography has revealed the effect of the control volume size and the correctness of the color scale setting, especially in the heterogeneous objects, on the results of elastometry. Methodological techniques to reduce the influence of subjective factors have also been proposed.

**Conclusion:**

The study has showed the possibility of using the modulus of stiffness difference between object and environment as a new criterion for comparative assessment of objects in shear wave elastometry taking into account stiffness of the environment. To reduce operator-dependence, it is necessary to take into consideration both the way of realizing elastometry (point or two-dimensional color elastography) and a number of other methodological factors.

## Introduction

Ultrasound shear wave elastography is a modern technique which allows one to quantitatively assess stiffness of soft biological tissues and to build their color image on the basis of measuring shear wave velocity for subsequent conversion to Young’s modulus [1, 2].

There are two ways of technical implementation of this method: point shear wave elastography and two-dimensional shear wave elastography. The point elastography makes it possible to measure only quantitatively the stiffness of the tissue in the selected local area, i.e. to perform elastometry. Two-dimensional elastography provides both planar visualization of the zone of interest in various shades of the color range corresponding to the stiffness of the scanned tissues and their subsequent quantitative assessment in any area of the obtained color elastogram [3, 4].

Two-dimensional image is achieved by creating several focused ultrasound, the so-called pushing pulses, sequentially generated at different depths with a small time delay. The wave fronts of these pulses form a widening cone (Mach cone) propagating in the tissues with the speed proportional to their stiffness; this cone may be traced using planar ultrasound waves with a high frame frequency [5].

Since there exists a diversity of ultrasound scanners implementing one or both elastographic techniques, there arises the question regarding accuracy and reproducibility of measurement results [6]. In this connection, a challenging task is to develop an optimal elastographic criterion for the comparative assessment of the objects with different stiffness in order to minimize the dependence of the results on the operator.

The reproducibility of the stiffness measurements is well evaluated on phantoms simulating soft biological tissue both with inclusions having a known Young’s modulus (calibrated phantoms) and with unknown stiffness of inclusions (uncalibrated phantoms) [7, 8]. The Verasonics research acoustic system with an open architecture (Verasonics Inc., USA), in which both methods of shear wave elastography are implemented, opens new opportunities for the comparative analysis [9].

**The aim of the study** is to develop a new criterion for the comparative assessment of objects with different stiffness during shear wave elastometry: a modulus of stiffness difference between object and environment.

## Materials and Methods

**Figure 1.**
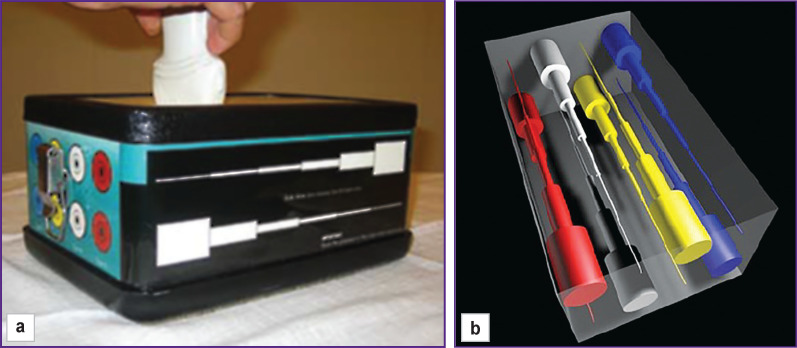
Calibrated phantom with cylindrical inclusions: (а) carrying out the study; (b) the scheme of inclusions in the phantom

At ***the first stage of the investigation*,** there was performed a point and two-dimensional shear wave elastography of the calibrated polymer Elasticity QA Phantom, model 049 (Computerized Imaging Reference Systems Company, USA) with homogeneous cylindrical inclusions of different diameters and stiffness degree located in the uniform matrix (Figure 1). In the grayscale scanning mode, all inclusions were homogeneous and isoechogenic with the surrounding background. Stiffness values obtained during elastometry on several ultrasound scanners were compared with each other and with nominal stiffness indicated in the documentation. The values of the modulus of stiffness difference between the objects and the environment have been compared.

The calibrated phantoms were fabricated from the Zerdine special polymer with the acoustic properties maximally approximated to the averaged physical parameters for human soft tissues: ultrasound wave propagation velocity was 1540±10 m/s, attenuation coefficient was 0.50±0.05 dB/cm.МHz, density — 1.04 g/cm^3^. The cylindrical inclusions located in the matrix have four types of stiffness (I–IV) with various known values of Young’s modulus.

Stiffness was measured in the cylinders of the greatest diameter (20 mm), which allowed us to avoid inclusion into the calculation zone of shear wave propagation the cylinders with a small diameter (>10 mm) [7]. The control volume was located in the center of the object, which made it possible to confirm with confidence the correctness of its stiffness measurement. An averaged value from five measurements was taken for subsequent analysis.

At ***the second stage of the investigation*,** BP1901 uncalibrated elastographic phantom (Blue Phantom, USA) was used; it represented a silicon model of the breast with rounded inclusions varying in echogenicity, stiffness, and degree of homogeneity randomly located in the acoustically uniform matrix. Stiffness of the inclusions was unknown and not indicated in the documentation. At first, localization, size, echogenicity, and uniformity of inclusions were evaluated using grayscale scanning. Thereafter, the quantitative values of their stiffness were determined by means of point and two-dimensional shear wave elastography.

An ultrasound investigation was carried out on three scanners with shear wave elastography technology: Aixplorer (SuperSonic Imagine, France) with SL15-4 linear sensor (4–15 МHz), Acuson S2000 (Siemens, Germany) with 9L4 linear sensor (4–9 МHz), and open-architecture Verasonics acoustic system (Verasonics Inc., USA) with L4-7 linear sensor and a specified operating frequency of 5 MHz. Aixplorer and Verasonics scanners are designed to perform point and two-dimensional shear wave elastography, while Acuson S2000 is for the point elastography only. While measuring the velocity by two-dimensional color elastogram on Verasonics and Aixplorer scanners, it was possible to change the diameter of the control volume from 1 to 10 mm, which influenced the quantitative values in the objects with non-uniform stiffness.

The Verasonics system is an ultrasound device designed to study elastic properties of various objects, to optimize technical processing of acoustic signals, and to develop the most informative setting modes for scanners. The chief advantage of the device is its openness, i.e. the possibility to change the parameters of the generated acoustic waves over a wide range including the application of shear wave elastography.

Construction of two-dimensional color elastogram is based on the excitation of several consecutive sources of shear wave propagation in the medium. For this purpose, a number of focused pulses creating shear waves are generated at different depths along the ultrasound beam. Five such pulses were used on the Verasonics. The originated shear waves interact with each other forming two quasi-planar waves propagating in different directions. The quasi-planar wave has a greater amplitude than an ordinary shear wave and therefore can travel sufficient distances and penetrate different objects in the medium, while its shape allows a uniform distribution over the entire depth of the examined area.

**Figure 2.**
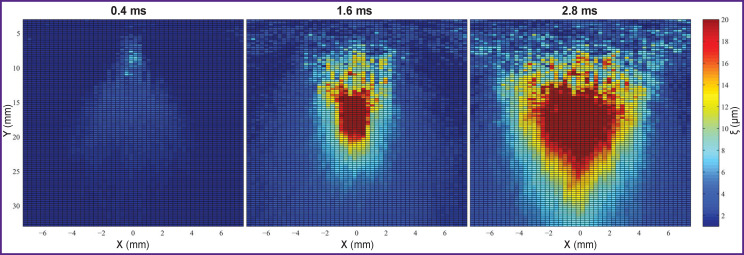
Visualization of formation and movement of quasi-planar shear waves at different time intervals

Once quasi-planar shear waves are generated, the process of registration of their propagation in the medium is carried out. For this purpose, a small sequence of imaging pulses is activated after each series of focused pulses. Their basic series is initiated already after the formation of quasi-planar waves and further propagation of these waves is registered. During data processing, displacement of the points in different coordinates of the examined area is calculated using correlation of one of the imaging pulses with the reference pulse. The wave delay caused by the medium displacement is determined by the shift of the correlation curve peak relative to zero. Calculation of the displacement allows one to visualize formation and propagation of the quasi-planar shear waves (Figure 2).

**Figure 3.**
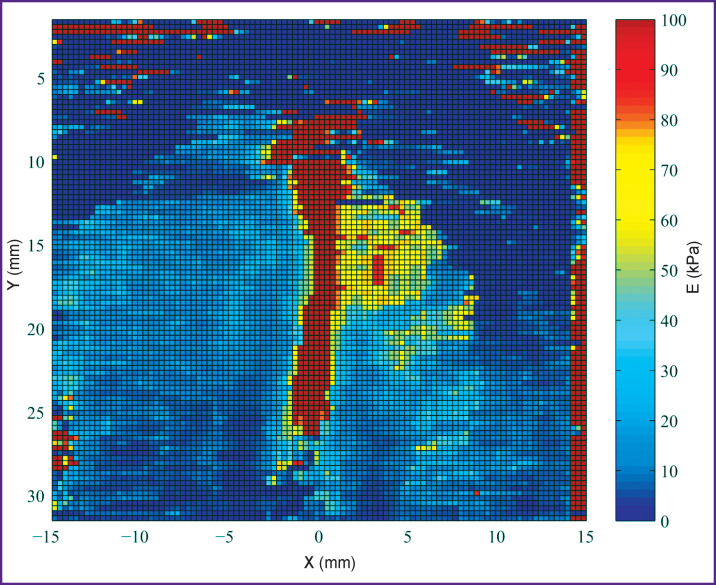
Color elasticity map for two-dimensional shear wave elastography on the Verasonics acoustic system

As a result, a map of elasticity of the examined area is obtained in the form of a matrix 30×30 mm or 101×101 points in size. Frame-by-frame recording of imaging pulses registering the propagation of the quasi-planar shear wave is used for its formation. At the time of its arrival, a clearly expressed maximum of particle displacement is fixed at each point of the medium. Comparing pixel coordinates along the axis parallel to the axis of quasi-planar shear wave propagation with the time of the wave arrival to the given point and knowing the same parameters for the neighboring pixel, it is possible to calculate the velocity of the wave on the given area. Eventually, the MatLab-based original program was used to create a map of Young’s modulus distribution depending on the object stiffness in the area under study (Figure 3).

Two-dimensional elastography technique works on the Aixplorer expert system according to the same principle. However, only the final result in the form of the ready color elasticity map is displayed for the user, with its superposition on a grayscale image of the examined area. The process of shear wave propagation itself in the medium is not visualized in this device.

## Results and Discussion

Since the Acuson S2000 scanner provides only point shear wave elastography and the Aixptlorer scanner performs point measurements using two-dimensional color elastogram, a comparative assessment of accuracy for both techniques was first done on the calibrated phantom to exclude the effect of technological causes on the results of measurements. We compared both numeric values of stiffness for these scanners with each other and with the data from the Verasonics scanner equipped with both elastometric options.

In order to assess the focus stiffness taking into account stiffness of the environment, we propose to use not only absolute values but also to introduce a relative quantitative indicator — a modulus of stiffness difference between focus and environment. It is more preferential to use the difference of absolute values than their ratio from the standpoint of the measurement error theory, and application of the modulus allows one to assess only quantitative difference between the stiffness of the focus and the medium. In this connection, all objects may be divided by the relative stiffness into comparable with the medium, differing from it slightly, moderately, and significantly. The modulus of stiffness difference

characterizes quantitatively the distinction between an object and a medium and reduces a well-known apparatus dependence in the evaluation of the shear wave elastometry results on the scanners from various manufactures since it is the difference of quantitative values taken into consideration during stiffness assessment, not the absolute figure.

Values of stiffness for the background and cylindrical inclusions, as well as moduli of their difference obtained by point shear wave elastography on the Verasonics and Acuson S2000 scanners, are presented in Table 1. Since the Acuson S2000 measures the shear wave velocity (*C*) without automatic conversion to the Young’s modulus (*E*), it was done manually using a known formula *Е*=3ρС^2^, where the medium density ρ was taken equal to 1 g/cm^3^.

**Table 1:** Stiffness of cylindrical inclusions and background, moduli of their stiffness difference

Measured object	Stiffness of inclusions and background		
	Certified value	Verasonics	Acuson S2000
***Average stiffness values (kPa)***			
Background, Eb	25.0	15.9	17.3
Cylinder 1, E1	8.0	5.1	6.4
Cylinder 2, E2	14.0	10.8	10.8
Cylinder 3, E3	45.0	32.7	32.3
Cylinder 4, E4	80.0	72.0	69.1
***Modulus of stiffness difference between inclusions and background***			
Eb–E1	17.0	10.8	10.9
Eb–E2	11.0	5.1	6.5
Eb–E3	20.0	16.8	15.0
Eb–E4	55.0	56.1	51.8

The results obtained are in line with those of previous studies on the calibrated spherical phantom [7] and show that all three ultrasound scanners (Verasonics, Acuson S2000, and Aixplorer) have a high and comparable accuracy of measuring shear wave velocity for stiffness determination. The study of stiffness difference moduli has demonstrated that all the devices during elastometry possess sufficient sensitivity allowing them to reveal small stiffness differences between the object and the background. The measuring accuracy does not depend on the technological implementation of elastometry — initially point elastometry or at the selected point on the two-dimensional color elastogram since these devices have one and the same physical base — generation of a shear wave and measurement of the velocity of its propagation in tissues.

**Figure 4.**
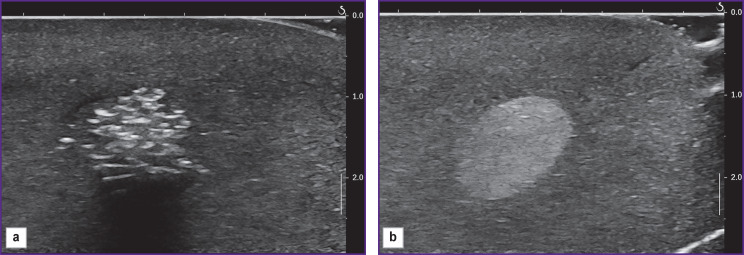
Examples of hyperechogenic inclusions 1 and 3 in the uncalibrated phantom: (а) non-uniform; (b) uniform

At the second stage of the investigation, an uncalibrated phantom was used, in which 11 objects with various acoustic properties were identified, of which 3 inclusions had a heterogeneous, mainly hyperechogenic, structure, the rest differed in echogenicity but were homogeneous (Figure 4).

Stiffness was measured for all inclusions (Table 2). Since the phantom was uncalibrated, it was thought to be impossible to judge the absolute accuracy of measurements performed by different scanners on the basis of the difference with specified values. Therefore, only comparative analysis of the obtained results was done including application of the modulus of difference between the identified inclusions.

**Table 2:** Stiffness of inclusions and background in uncalibrated phantom

**Measured object**	**Average stiffness values (kPа)**			
	Verasonics, point elastometry	Verasonics, two-dimensional elastometry	Acuson S2000	Aixplorer
Background, Eb	5.3	54.1	52.2	75.0
***Non-uniform structure***				
Inclusion 1, Е1	147.8	144.7	149.1	171.2
Inclusion 5, Е5	158.9	165.7	189.1	179.8
Inclusion 11, Е11	153.4	144.1	163.4	179.0
***Uniform structure***				
Inclusion 3, Е3	39.4	55.7	48.7	67.1
Inclusion 4, Е4	32.7	64.0	56.2	70.0
Inclusion 6, Е6	43.6	57.1	44.9	77.8
Inclusion 7, Е7	34.4	40.4	52.7	68.1
Inclusion 10, Е10	62.9	65.3	51.9	79.9
***Unechogenic structure***				
Inclusion 2, Е2	—	2.4	—	1.4
Inclusion 8, Е8	—	5.1	—	3.5
Inclusion 9, Е9	—	3.2	—	2.3

**Figure 5.**
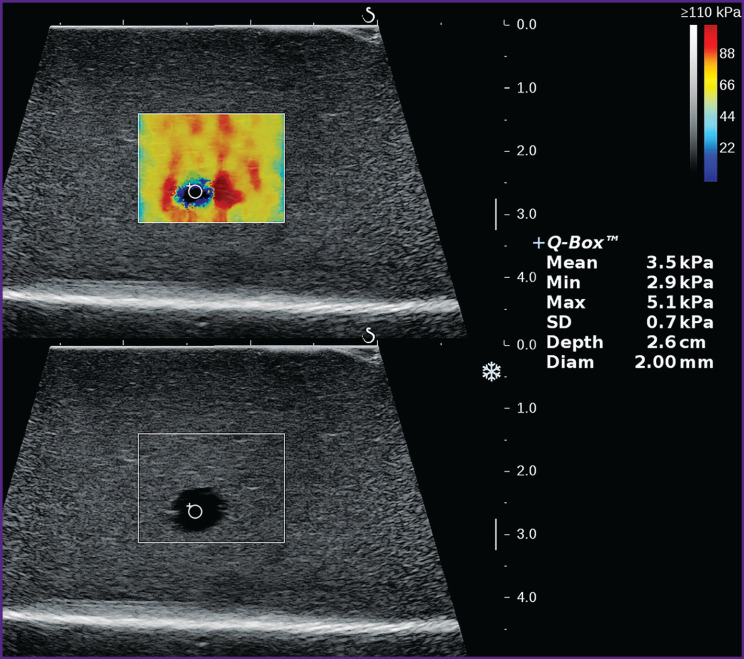
Elastogram of unechogenic cyst-like inclusion showing values of content stiffness (echograms from the Aixplorer scanner)

While measuring the stiffness of unechogenic cyst-like inclusions using point elastography, no results were obtained, but measurements performed by two-dimensional color map elastography provided regularly quantitative values. It is difficult to judge reliably the reason for such discrepancy between the methodologies: it is most likely that the device registers low-amplitude noise signals, which are of no diagnostic value (Figure 5).

**Figure 6.**
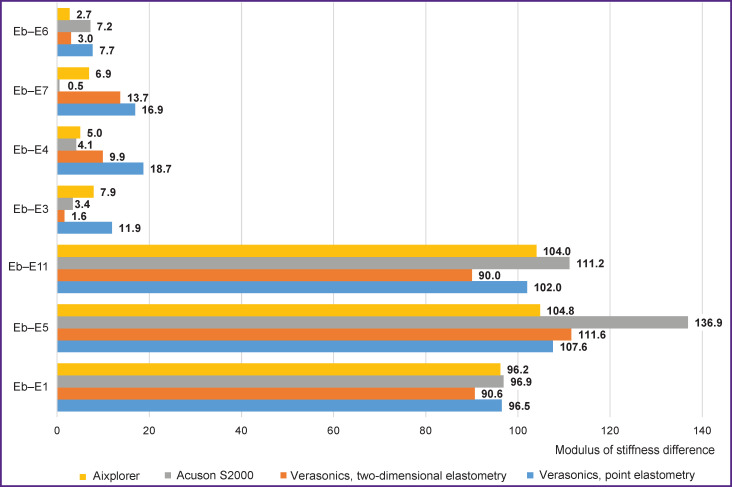
Moduli of stiffness difference between inclusions and background in the uncalibrated phantom

High values of the modulus of stiffness difference characterize considerable distinction from the stiffness of the object and environment, which evidences explicitly the presence of a focus (Figure 6). When the modulus of stiffness difference between the examined area and the environment is insignificant, a question arises whether to interpret this area as a non-uniformity of the media itself or a focal formation comparable with it in stiffness. A considerable difference of their echogenicity in the grayscale imaging and well-defined boundaries speak in favor of a focus, but in case of practically isoechogenic ratio and unclear boundaries the question remains open and may be answered only by contrast-enhanced ultrasound examination.

But there is another question: what is permissible non-uniformity of the biological medium, for example, the cyrrotically changed liver parenchyma, to show the ultimate modulus of stiffness difference which, when exceeded, will denote the area of non-uniformity as a focal formation? The answer requires a further in-depth study.

**Figure 7.**
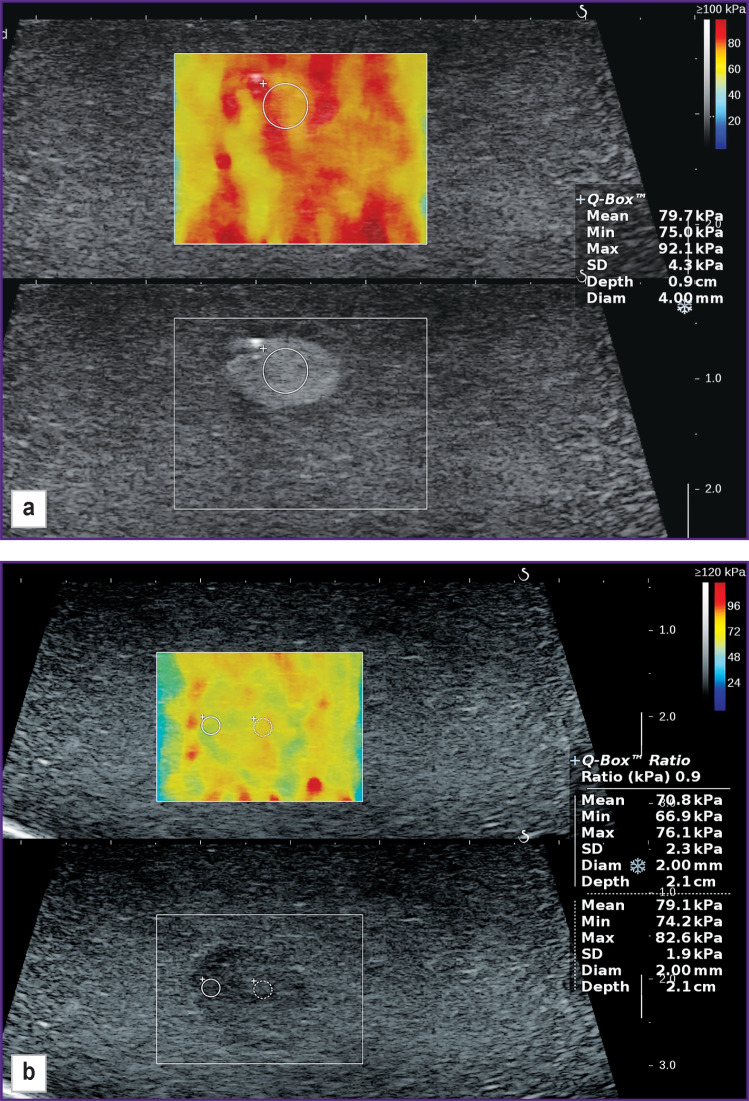
Object with similar stiffness not visualized on color elastogram (echograms from the Aixplorer scanner): (а) hyperechogenic formation; (b) isoechogenic formation

Figure 7 presents formations of various echogenicity but having stiffness similar to the medium and not discernible on color elastograms. The inclusion in Figure 7 (a) is easily visualized in the grayscale mode, while inclusion in Figure 7 (b) may be missed in both the grayscale and elastographic image.

It has been established that during elastometry using two-dimensional elastograms, the size of the control volume strongly influences local values of stiffness of hyperechogenic heterogeneous inclusions, as well as their distinction from the environment. So, if its diameter was 1 mm, the average values of local stiffness in different inclusion areas on the Aixplorer scanner had significant variations from 111 to 297 kPa, whereas at larger diameters (up to 4 mm), the spread in values reduced considerably, and at 7 mm it remained reproducible within the measurement error. In homogeneous inclusions, such a marked effect of the control volume size on the difference of the local stiffness values from the average value for the entire inclusion has not been noted.

**Figure 8.**
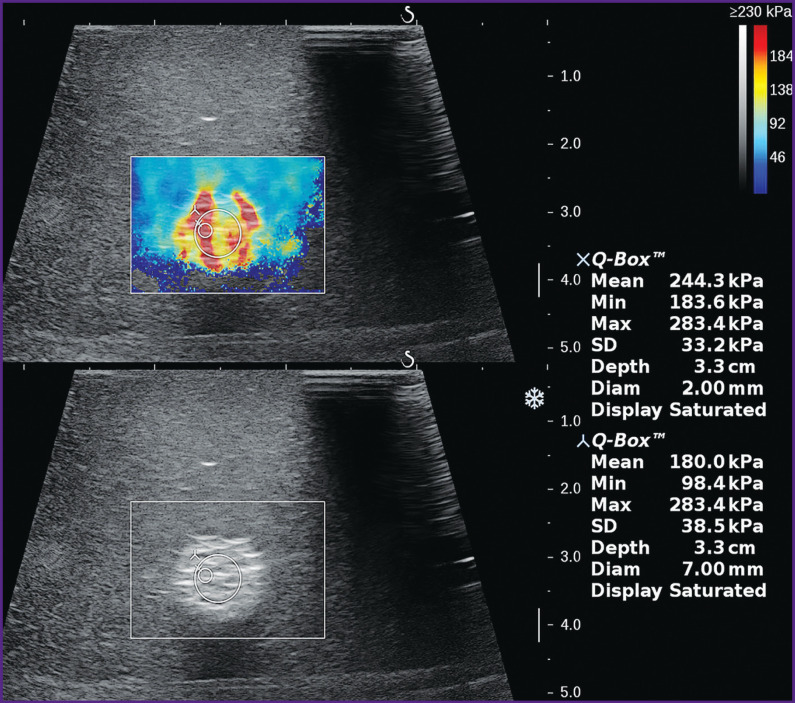
Effect of the control volume size (Q-Box) on the inclusion stiffness value (echograms from the Aixplorer scanner)

The correct choice of the control volume is especially important when small objects of 10–12 mm in diameter are examined. In this case, there exists the likelihood of the total measurement of the shear wave velocity in the object and beyond it, which may result in the distortion of the numerical values, especially if the difference of the absolute stiffness values between the object and environment is considerable [7]. Performing elastometry by two-dimensional color elastogram, the size of the control volume should be selected in such a way that it occupies the entire central part of the formation, whereas several millimeters of the examined object tissue are left between it and the boundary of the formation (Figure 8). In the absolute figures, the diameter of the control volume must be not less than 6 mm, which corresponds to the size of the unchanged control volume when point elastometry is done on the scanners of other manufacturers.

Upon the whole, values of stiffness for the background and inclusions obtained on different scanners are comparable, except for the Aixplorer, whose data were systematically higher than the results of investigation on two other devices. Comparing the stiffness measurements performed on the Verasonics using point elastometry and two-dimensional color elastometry, a certain difference of the results was noted in the echogenically homogeneous inclusions expressed in smaller values of stiffness for point elastometry. Since the process of determining the shear wave velocity in the Verasonics is technically the same, operator-dependent measurements may be considered the reason for this difference. The influence of the subjective factor on the elastometric accuracy is now being studied and discussed in the world literature, and the results of these investigations are not so unambiguous [10, 11].

During point elasometry, the physician determines the velocity of the shear wave randomly in various areas of the grayscale image of the object without having any color image of its stiffness. When measuring the local stiffness using a ready two-dimensional color elastogram, the physician immediately sees in color the stiffness differences of the object, which can influence the choice of the areas for measurement and final values. The argument in favor of this may be the fact that the results of measuring the uniform objects on the Verasonics using point elastometry appeared to be closer to the data obtained on the Acuson S2000, while a separately conducted study using two-dimensional color elastogram showed the results closer to those obtained on the Aixplorer. In the non-uniform inclusions, the results seemed to be more comparable with each other due to a greater number of measurements in the areas with various echogenicity, which resulted in averaging of the results of both methods with leveling of the difference between them.

**Figure 9.**
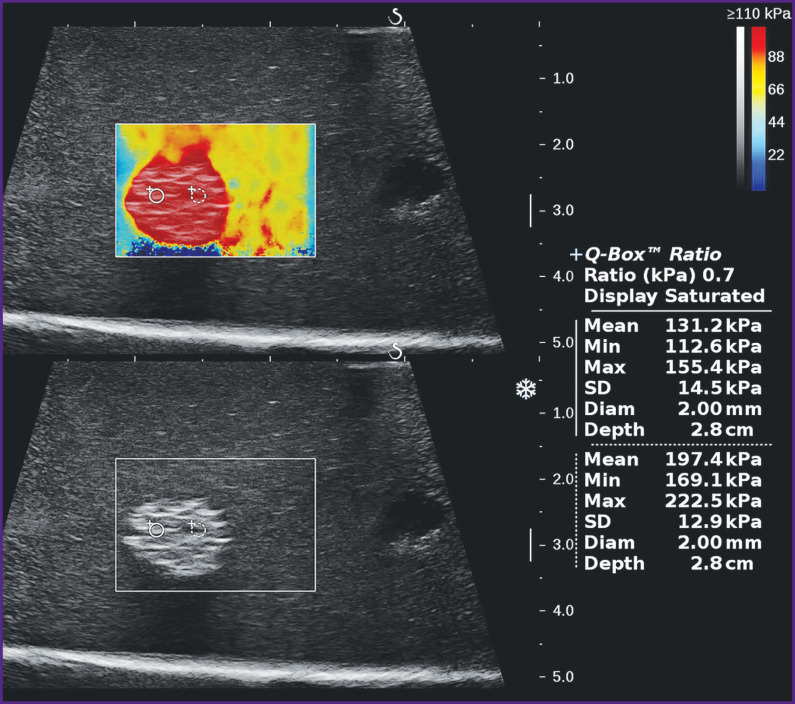
Effect of color stiffness scale adjustment on the choice of zones for point elastometry (the same inclusion as in Figure 8) With an understated scale, a non-uniform inclusion acquires almost homogeneous red color, but the correctly set maximal value of the color scale provides good visualization of different inclusion stiffness (echograms from the Aixplorer scanner)

In two-dimensional elastography of a non-uniform focus or medium, an important factor of the measuring correctness and subsequent comparison is the correct adjustment of the color stiffness scale to the greatest value (in kPa or m/s) to minimize the subjective factor in the selection of the zones for point elastometry. This correctly assigned value of the stiffness scale must exceed insignificantly maximal values for the given medium or object obtained during initial experimental measurements and should be displayed in the most intensive red color. This gives an extremely wide color range of stiffness imaging and reduces the subjective factor in selecting the areas for point elastometry. If the greatest value on the scale is set too low, the areas with the smaller real stiffness value will be colored in intensive red, creating an illusion of a uniform, very stiff focus, leading to the incorrect choice of the zone for point elastometry (Figure 9).

## Conclusion

The results of the conducted experimental study allowed us to propose a new criterion for comparative elastomeric assessment — the modulus of stiffness difference between object and medium. The methodological aspects of its application have been developed. In order to reduce operator-dependence, it is suggested taking into account the way of elastometry implementation (point or two-dimension color elastography), as well as other factors: control window size, adjustment of the stiffness scale, the number of measurements, their distribution in the object.

## References

[1] Sarvazyan A.P., Rudenko O.V., Swanson S.D., Fowlkes J.B., Emelianov S.Y. (1998). Shear wave elasticity imaging: a new ultrasonic technology of medical diagnostics.. Ultrasound Med Biol.

[2] Sarvazyan A.P., Rudenko O.V., Fatemi M. (2021). Acoustic radiation force: a review of four mechanisms for biomedical applications.. IEEE Trans Ultrason Ferroelectr Freq Control.

[3] Shiina T., Nightingale K.R., Palmeri M.L., Hall T.J., Bamber J.C., Barr R.G., Castera L., Choi B.I., Chou Y.H., Cosgrove D., Dietrich C.F., Ding H., Amy D., Farrokh A., Ferraioli G., Filice C., Friedrich-Rust M., Nakashima K., Schafer F., Sporea I., Suzuki S., Wilson S., Kudo M. (2015). WFUMB guidelines and recommendations for clinical use of ultrasound elastography: part 1: basic principles and terminology.. Ultrasound Med Biol.

[4] Mitkov V.V., Mitkova M.D. (2015). Ultrasound shear wave elastography.. Ul’trazvukovaa i funkcional’naa diagnostika.

[5] Rudenko O.V., Safonov D.V., Demin I.Yu., Rykhtik P.I., Andreev V.G., Gurbatov S.N., Romanov S.V., Glava 1., Borsukov A.V. (2017). Shear wave elastography: an analysis of clinical cases]..

[6] Dietrich C.F., Bamber J., Berzigotti A., Bota S., Cantisani V., Castera L., Cosgrove D., Ferraioli G., Friedrich-Rust M., Gilja O.H., Goertz R.S., Karlas T., de Knegt R., de Ledinghen V., Piscaglia F., Procopet B., Saftoiu A., Sidhu P.S., Sporea I., Thiele M. (2017). EFSUMB guidelines and recommendations on the clinical use of liver ultrasound elastography, update 2017 (long version).. Ultraschall Med.

[7] Safonov D.V., Rykhtik P.I., Shatokhina I.V., Romanov S.V., Gurbatov S.N., Demin I.Yu. (2017). Shear wave elastography: comparing the accuracy of ultrasound scanners using calibrated phantoms in experiment.. Sovremennye tehnologii v medicine.

[8] Kulberg N.S., Osipov L.V., Usanov M.S. (2016). Comparative analysis of the elastography technologies in ultrasonic diagnostic devices using elastography phantom.. Radiologia – praktika.

[9] Khalitov R.S., Gurbatov S.N., Demin I.Y. (2016). The use of the Verasonics ultrasound system to measure shear wave velocities in CIRS phantoms.. Phys Wave Phenom.

[10] Yu Y., Xiao Y., Cheng J., Chiu B. (2018). Breast lesion classification based on supersonic shear-wave elastography and automated lesion segmentation from B-mode ultrasound images.. Comput Biol Med.

[11] Chamming’s F., Hangard C., Gennisson J.L., Reinhold C., Fournier L.S. (2021). Diagnostic accuracy of four levels of manual compression applied in supersonic shear wave elastography of the breast.. Acad Radiol.

